# Noninvasive Non-Contact SpO_2_ Monitoring Using an Integrated Polarization-Sensing CMOS Imaging Sensor

**DOI:** 10.3390/s22207796

**Published:** 2022-10-14

**Authors:** Mukul Sarkar, Maher Assaad

**Affiliations:** 1 Electrical Engineering Department, IIT Delhi, Hauz Khas, New Delhi 110016, India; 2 Department of Electrical and Computer Engineering, Ajman University, Ajman P.O. Box 346, United Arab Emirates

**Keywords:** polarization, CMOS image sensor, imaging sensors, wire grid polarizers, oximeter, pulse oximeter, SpO_2_ measurements

## Abstract

Background:In the diagnosis and primary health care of an individual, estimation of the pulse rate and blood oxygen saturation (SpO${}_{2}$) is critical. The pulse rate and SpO${}_{2}$ are determined by methods including photoplethysmography (iPPG), light spectroscopy, and pulse oximetry. These devices need to be compact, non-contact, and noninvasive for real-time health monitoring. Reflection-based iPPG is becoming popular as it allows non-contact estimation of the heart rate and SpO${}_{2}$. Most iPPG methods capture temporal data and form complex computations, and thus real-time measurements and spatial visualization are difficult. Method:In this research work, reflective mode polarized imaging-based iPPG is proposed. For polarization imaging, a custom image sensor with wire grid polarizers on each pixel is designed. Each pixel has a wire grid of varying transmission axes, allowing phase detection of the incoming light. The phase information of the backscattered light from the fingertips of 12 healthy volunteers was recorded in both the resting as well as the excited states. These data were then processed using MATLAB 2021b software. Results: The phase information provides quantitative information on the reflection from the superficial and deep layers of skin. The ratio of deep to superficial layer backscattered phase information is shown to be directly correlated and linearly increasing with an increase in the SpO${}_{2}$ and heart rate. Conclusions: The phase-based measurements help to monitor the changes in the resting and excited state heart rate and SpO${}_{2}$ in real time. Furthermore, the use of the ratio of phase information helps to make the measurements independent of the individual skin traits and thus increases the accuracy of the measurements. The proposed iPPG works in ambient light, relaxing the instrumentation requirement and helping the system to be compact and portable.

## 1. Introduction

Noninvasive measurements of the pulse rate and arterial oxygen saturation (SaO${}_{2}$) content of the blood are important for real-time health monitoring. The arterial blood oxygen saturation is defined as the fraction of oxygen-saturated hemoglobin relative to total hemoglobin in the arterial blood. This indicates the patient’s oxygenation status and is vital for the early detection of hypoxemia. The gold standard for measurement of the oxygen saturation is the arterial blood gas (ABG) [1]. ABG measures the balance of oxygen and carbon dioxide in the blood. Aside from the oxygen saturation in the arterial blood, ABG also helps in determining the partial pressure of oxygen (PaO${}_{2}$) and total oxygen concentration. The point-of-care ABG analyzers typically measure PaO${}_{2}$ to calculate the oxygen saturation, which for patients with abnormal hemoglobin results in wrong measurements. Using ABG, the oxygen saturation is known for only one point in time, and therefore, it is difficult to state whether the oxygen saturation is transiently low or if it is continuously low. In health care monitoring units, the averaging of oxygen saturation and trends is important. Further, ABG is invasive, painful, time-consuming, costly, and impractical in most primary health care settings.

Pulse oximeters are used for noninvasive estimation of arterial hemoglobin oxygen saturation. Pulse oximeters [2] are the most popular instruments used to simultaneously measure the heart rate and SpO${}_{2}$. SpO${}_{2}$ is an indirect estimation of the arterial oxygen saturation (SaO${}_{2}$). The arterial oxygen saturation (SaO${}_{2}$) represents the percentage of binding sites on hemoglobin carrying oxygen. SpO${}_{2}$ measures the amount of oxygen available by measuring the binding sites with oxygen. The pulse oximeter detects the amount of oxyhemoglobin and deoxygenated hemoglobin in arterial blood and presents it as oxyhemoglobin saturation (SpO${}_{2}$). Pulse oximetry has become a standard in primary and intensive care units to determine the limit of cardiopulmonary stress characterized by the decrease in oxygen saturation in the blood. Pulse oximetry has been widely used in rehabilitated COVID-19 patients and recommended by the World Health Organization for self-monitoring.

Pulse oximetry is an optical-based method for estimating SpO${}_{2}$ by using the photoplethysmography (PPG) waveform for two different wavelengths. The wavelengths are chosen to provide the differential absorption characteristics of oxyhemoglobin and deoxyhemoglobin. A pulse oximeter using PPG to evaluate peripheral blood oxygen saturation uses the red and infrared light absorption levels. Pulse oximeters can either use transmission-based sensors or reflection-based sensors. In transmission-based sensors, the light emitter and detector are on the opposing sides of the measurement site. Here, the light from the emitter is partially absorbed by the body tissue, while the remaining light reaches the detector. Transmission-based sensors are suitable for fingers, toes, or earlobes [3]. In reflection mode, the light emitter and the detector are adjacent to one another [4]. The incident light is reflected or backscattered from various depths underneath the skin. The pulse rate and SpO${}_{2}$ are determined from the absorbed (transmission-based) or reflected (transmission-based) intensity measured by the detector. The noninvasive and continuous measurements of arterial blood oxygen saturation using pulse oximetry gained popularity due to their low cost, simple structures, and practicality.

Transmission-based pulse oximeters are mostly contact-based devices and are used in peripheral areas, such as the fingers and ears. An intensity-based plastic optical fiber sensor for noninvasive monitoring of the carotid pulse waveform is presented in [5]. In contact-based devices, the measurements are extremely sensitive to the subject’s movements of his or her fingertips. In [5], the movements of the subjects and the operator change the reflectors’ and sensor’s alignments, resulting in variations in the acquired signal intensity. It becomes more challenging to monitor the pulse rate in infants due to the constant movement of the fingertips. Transmission-based methods are suitable for body sites with thick tissue, such as the index fingers, while reflection-based methods are applicable on most body sites [6]. Reflective-mode pulse oximetry, or imaging photoplethysmography (PPG), is an imaging-based solution for determining the pulse rate and SpO${}_{2}$ without direct skin contact [7].

Imaging photoplethysmography (iPPG) allows the use of diverse measurement sites such as the feet, forehead, chest, and wrists, in addition to the fingertips, in a non-contact and noninvasive manner [8]. In a multi-site PPG system, PPG signals are measured simultaneously from the finger, toe, and earlobe. The PPG signals from different body sites have different signal qualities [9] and waveform shapes [10]. The PPG waveforms are influenced by breathing patterns. Deep breathing enhances the amplitude fluctuations in the PPG signal compared with normal or slow breathing [11]. A quantitative investigation of the effects of breathing patterns and the measurement sites on the PPG waveform was studied in [12]. Here, PPG signals from different measurement sites were recorded under resting and deep breathing patterns. It was concluded that the fingers and earlobe are relatively better measurement sites for PPG analysis. The fingers and earlobes have rich arterial supplies, and their accessibility makes them reliable measurement sites for arterial pulsation [13,14]. The spatial redundancy of the camera sensor used in the imaging device increases the robustness of the measurements compared with single-spot measurements by the contact-based pulse oximeter.

The imaging PPG is classified into active and passive systems. In an active imaging system, PPG is performed with two different wavelengths, typically red (660 nm) and infrared (IR) (940 nm). In [15], smartphone-based, non-contact imaging photoplethysmography was used to accurately estimate the SpO${}_{2}$ level. The illumination source is the built-in flashlight of the mobile phone. The ratio of the intensities measured from the green and red channels is used to obtain a multiple linear regression to improve the SpO${}_{2}$ estimation. The algorithm obtains the desired ratios from the reflectance images recorded by the RGB channels and sets appropriate weights in the calibration process for the determination of the SpO${}_{2}$. The SpO${}_{2}$ was estimated for five healthy volunteers with an estimated 0.029 ± 1.141% error between the smartphone and the reference oximeter. Active imaging PPGs are highly sensitive to ambient light sources, which act as noise and reduce the accuracy of the measured data. In [15], to reduce the specular reflection from the skin surface, a polarizer sheet at the output of the flashlight and an analyzer sheet in front of the camera was used. In [16], SpO${}_{2}$ was extracted using a dual-wavelength light-emitting diode array of 760 and 880 nm and a CMOS detector. The active measurement set-ups require a light source close to or in contact with the skin and are therefore not helpful in non-contact measurements. Furthermore, the ambient light always acts as a source of noise, degrading the measurements.

In a passive imaging system, the PPG signal is acquired by the camera, with ambient light as the illumination source [17]. A non-contact method for blood saturation measurement using a double CCD, each with a narrow bandpass filter and ambient light as a lighting source, is presented in [18]. The bandpass filters block the ambient light and thus reduce the interference with other bands of interest. The PPG signals were measured remotely on the human face with ambient light and a consumer-level photo camera in [19]. In this work, the pulse rate was detected even at a distance of 1.5 m. A face imaging-based heart rate monitoring technique was proposed in [20]. The reflectance image from the face is decomposed, and the heart rate is evaluated based on the periodic variation in reflectance strength. The reflectance changes due to variations in the absorption of different wavelengths by hemoglobin molecules as the heart rate changes. An ensemble empirical mode decomposition (EEMD) of the Hilbert–Huang transform (HHT) is used to acquire the heart frame from the images captured while reducing the effect of the ambient light. The passive measurement systems are not limited by the distance between the detector and the fingertip.

In a passive imaging-based system, the ambient light often saturates the camera sensor and reduces the accuracy of the measurements. The reflection of the light from the skin depends on the skin’s color and texture. This makes the measurement individual-specific. Dark skin has a higher content of melanin, and therefore, the quality of the reflected signal is affected, resulting in overestimation of the SpO${}_{2}$ level. Furthermore, the calibration required is also cumbersome due to diffusion of the reflected light that has traveled to much shallower tissue depths over much smaller distances of the fingertip. In [21], accurate measurements were obtained even during significant subject motion by exploiting prior signatures in the amplitudes at different wavelengths. This reduces the noise in the measurement data. However, the calibration and processing make it difficult to operate in real time.

To make the measurements less susceptible and robust, reflective polarization (phase-based) measurements are used. There have been several studies in the literature leveraging polarization to improve the accuracy of pulse oximetry or PPG. In phase-based measurements, the phase characteristics of the backscattered light when the incident light is linearly polarized are studied. For phase detection, an optical test bench with a camera system and polarization filters are used. A collimated optical test bench with a radially polarized, incoherent LED-based light source was used to estimate the SpO${}_{2}$ level in [22]. Here, a collimated optical test bench was used with an incoherent light source. The incident light was polarized using external linear polarizers. The SpO${}_{2}$ level was measured for six different individuals with varying skin tones under normal breathing conditions while being seated. The experimentally obtained SpO${}_{2}$ level was claimed to be more consistent than those determined by the reference pulse oximeters (Metene as well as a medical-grade pulse oximeter (Masimo)).

In [23], the depolarized or deep component of the reflected light is used to observe the relative oxygen saturation. However, the SpO${}_{2}$ measurements were dependent on the skin texture, color, and other characteristics. To negate the influence of skin color, the specularly reflected light was used to normalize the reflection from the superficial and deep capillary vessels in [24,25]. The SpO${}_{2}$ level varied from 93% to 99% by varying the breathing pattern in 15 healthy subjects. The backscattered light from the fingertip was used to determine the light components reflected from the deep and superficial layers of the skin. This ratio was shown to be linearly correlated with the SpO${}_{2}$ levels. The specularly reflected light component reduced the accuracy of the blood pulsation measurements. Cross-polarization was used to remove the specular component to maximize the reflected signal from the tissue and thus increase the accuracy of the SpO${}_{2}$ reading. Polarization-sensitive PPG (PSPPG) removes the specular components and increases the accuracy of the blood pulsation measurement over conventional PPG by 30%, as presented in [26]. The use of external filters requires multiple sequential measurements by manually varying the transmission axis of the external polarizer to estimate the SpO${}_{2}$ level. The measurements are thus susceptible to variations in the transmission axis of the external polarizers, reducing the accuracy of the measurement.

In this paper, a reflective mode iPPG is proposed using a custom complementary metal oxide semiconductor (CMOS) image sensor with a wire grid polarizer on every pixel. The embedded wire grid polarizer on each pixel helps with eliminating the measurement errors inherent in passive polarization-based systems. The use of ambient light removes the necessity of using a dedicated light source, thus making the device more compact. The ratio of the reflected light from the deep and superficial layers of the skin is correlated to the pulse rate and SpO${}_{2}$ measurements. The ratio is monotonically linear with an increasing SpO${}_{2}$ level. The measurements are carried out with volunteers in both resting and excited states. The simultaneous capture of phase information with multiple transmission axes allows for snapshot measurements of the heart rate and SpO${}_{2}$ level. The reduction in the temporal data to be processed for SpO${}_{2}$ estimation allows faster computation and application in real time. The quick determination of the change in state from resting to excited or vice versa helps in making appropriate medical decisions.

The rest of the paper is organized as follows. Section 2 describes the theory of polarization-based pulse rate and SpO${}_{2}$ measurements. The working of the pulse oximeter is presented in Section 2.1. Section 2.2 describes the optical sectioning of the skin under the fingertip and the nature of the backscattered light from the deep and the superficial layers. Section 3 describes the experimental set-up. The custom-designed CMOS image sensor with a wire grid polarizer is briefly described in Section 3.1. The data collection methodology and analysis are explained in Section 3.2 and Section 3.3, respectively. Section 4 presents the measurement results and their analysis, and Section 5 concludes this paper.

## 2. Methodology

### 2.1. Pulse Oximetry

The measurement of oxygen saturation in an individual’s blood provides preliminary information about the cardiovascular as well as the respiratory systems. A healthy person has an oxygen saturation of 95–100%. The pulse oximeter is useful for estimating the amount of oxygen in the blood without requiring a blood sample. Pulse oximeters are optical sensors with two LEDs and a photodiode placed on a thin part of the patient’s anatomy, such as a fingertip. The two LEDs are paired and transmit light alternately with different wavelengths, which travel through the fingertip and are detected by a photodiode on the opposite side. The selection of the wavelength for the pulse oximeter follows two principles. First, the absorption coefficients of HbO${}_{2}$ and Hb at one wavelength should differ greatly. The second is an approximately equal absorption coefficient in terms of HbO${}_{2}$ and Hb at the other wavelength [18]. The conventional oximeter generally uses LEDs with wavelengths of 660 nm and 940 nm.

Hemoglobin that does not contain O${}_{2}$ (deoxyhemoglobin) shows high absorption of LED red (660-nm) light and, hemoglobin that contains O${}_{2}$ (oxyhemoglobin) has high absorption of infrared (940-nm) light. The oxygen saturation is obtained by calculating the ratio between the absorption of the two wavelengths. The value of the SpO${}_{2}$ level can be calculated using the expression

(1) $${\mathrm{SpO}}_{2}=\frac{{\mathrm{HbO}}_{2}}{\mathrm{Hb}}+{\mathrm{HbO}}_{2}$$

where SpO${}_{2}$ is the oxygen saturation, HbO${}_{2}$ is the oxygenated hemoglobin, and Hb is the non-oxygenated hemoglobin.

Pulse oximeters have limitations and the risk that an inaccurate measurement may result in unrecognized low oxygen saturation levels. The commercially available over-the-counter pulse oximeter accuracy is highest at saturations of 90–100%, intermediate at 80–90%, and lowest below 80%. SpO${}_{2}$ measurement variation in terms of accuracy and its dependence on the individual traits provides more utility for trends over time instead of absolute thresholds.

### 2.2. Optical Sectioning of the Skin on a Finger Tip

The optical behavior of the human blood is complex, primarily due to the highly concentrated hemoglobin content. The light is absorbed and scattered by the discoid-shaped erythrocytes. Hemoglobin has distinct absorption peaks in the visible range and is not independent of scattering. It is, therefore, difficult to describe the optical behavior using the Mie theory, which is used in highly diluted blood in spectral ranges of 600–1100 nm, where the hemoglobin absorption is low. The limitation of the Mie theory in describing the optical properties of blood is overcome by the use of transport equations. As per the transport theory, the optical properties of blood can be described by the intrinsic optical parameter absorption coefficient $\mu_{a}$, scattering coefficient $\mu_{s}$, and anisotropy factor *g* [27]. The transport theory takes into consideration the non-spherical shape of the red blood cells, the phenomenon of coupled absorption and scattering, multiple scattering, and interference phenomena between close neighboring cells and inhomogeneous local distribution within the flow stream. Accordingly, the reflected light is dependent on the optical path length and the concentration of $O_{2}$ in the blood vessels.

The elastic deformations of the capillary bed due to pulse oscillations of arterial transmural pressure change the light absorption and scattering coefficients. Therefore, for monitoring arterial pulsations, deep penetration of the light source is not required. The oxygenated blood containing HbO${}_{2}$ flows in vessels that are situated relatively deeper compared with the vessels carrying deoxygenated blood (Hb) [28]. The light penetration depth into the skin is usually defined as a path on which the light intensity is decayed to the level of 1/e [28]. The absorption is thus higher in the capillary bed with HbO${}_{2}$ compared with Hb. The absorption of the scattering particles significantly influences the anisotropy factor. The increase in the complex refractive index increases the reflection while decreasing the transmittance. This results in increased backscattering of the incident photons. The backscattering is higher for light reflected from the capillary bed containing Hb compared with HbO${}_{2}$.

**Figure 1:**
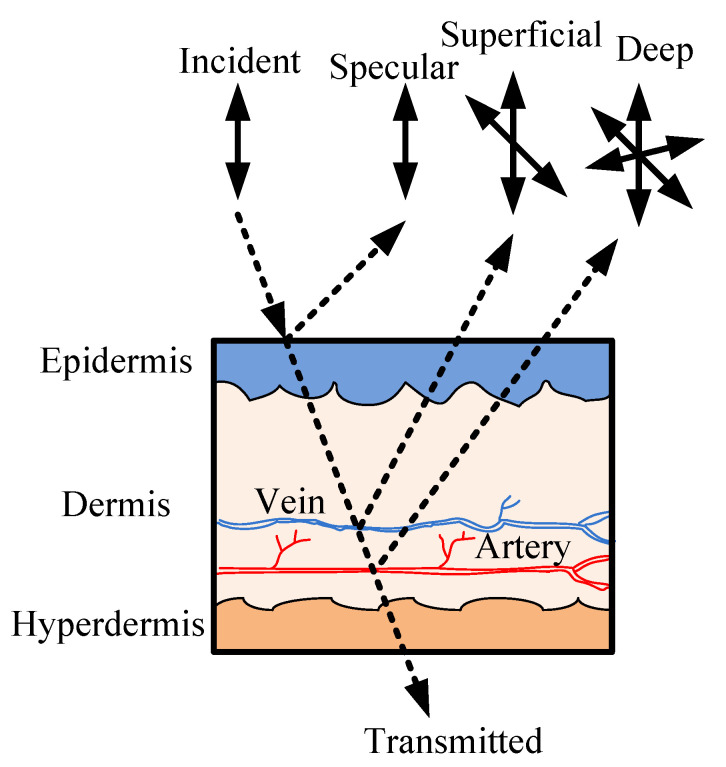
Backscattering profile of the skin at a finger tip [24].

The variation in the scattering from the fingertip is shown in Figure 1 [24]. As the polarized or unpolarized light enters the skin, there is a specular reflection from the surface itself. Aside from the specular reflection, there is a superficial reflection from the upper capillary bed and a deep reflection from the lower capillary bed. The specularly reflected light mostly retains the incident polarization state. The optical path length for deep scattering is higher compared with superficial scattering. Therefore, the depolarization is also greater. as shown in Figure 1. A characteristic relationship between the deep and superficial scattering and the SpO${}_{2}$ level can be expressed as

(2) $${\mathrm{SpO}}_{2}=k_{1}\times \left(\frac{I_{deep}}{I_{superficial}}\right)+k_{2}$$

where $k_{1}$ and $k_{2}$ are two constants that can be obtained empirically while $I_{deep}$ and $I_{superfical}$ are the backscattered intensities of the light. The differential backscattering is used in this work to estimate the SpO${}_{2}$ level. The difference in backscattering is found using the phase of the reflected light. The increase in the backscattering results in more depolarization and therefore lowers the degree of polarization (DoP). The degree of polarization measures the extent of depolarization in the backscattered light.

## 3. Experimental Set-Up

**Figure 2:**
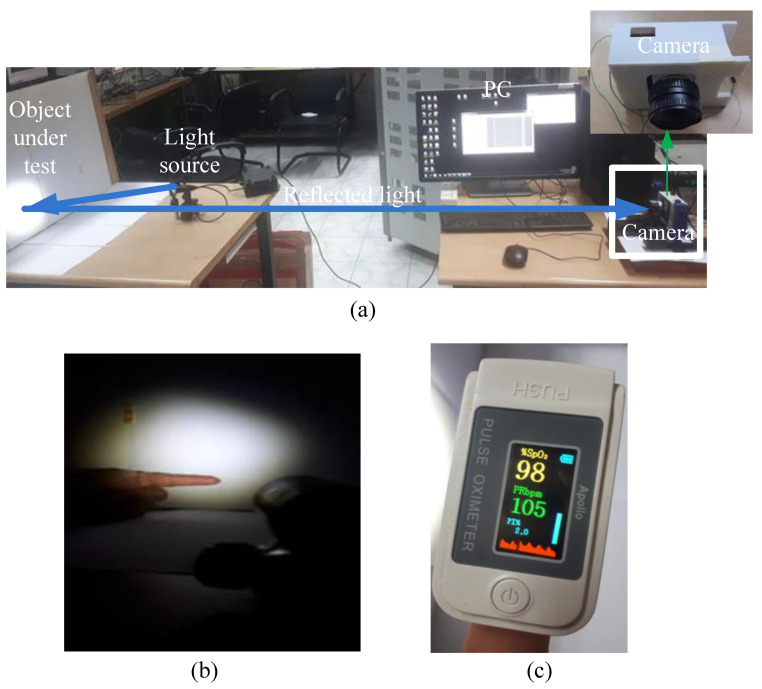
(**a**) The experimental set-up, (**b**) the position at which the image of the fingertip is captured, and (**c**) the commercial oximeter used for reference.set-up.

The experimental set-up is shown in the Figure 2a. The optical test bench includes a DC light source from Holmarc Mechtronics Pvt. Ltd. representing the background light. The light source was adjusted to provide uniform illumination over the entire region of interest on the fingertip. The light source generated unpolarized light which was either directly incident on the test subject or polarized using external polarization filters (ED-P-02-PRM, from Holmarc Mechatronics Pvt. Ltd., Kochi, Kerela, India). The incident light was reflected from the object under testing and captured by a custom-designed monochromatic camera. The positions of the fingertip and the incident light are shown in Figure 2b. For experimental purposes, the environment chosen was dark to prevent saturation of the camera sensor from background light. The images captured by the custom camera were processed using a commercial software tool. For reference, a commercial pulse oximeter (Figure 2c) from Apollo was used. The commercial oximeter was placed on the finger of the other hand, and reference measurements were noted for all experiments.

### 3.1. Custom Image Sensor with a Wire Grid Polarizer on Each Pixel

**Figure 3:**
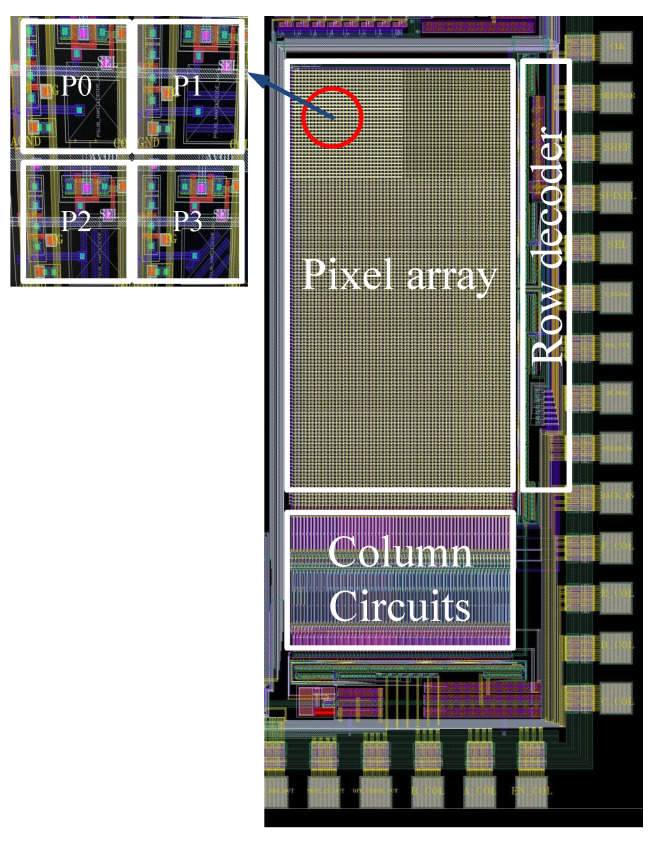
Microchip photograph showing pixels with wire grid polarizers.

The custom-designed camera used a CMOS image sensor with an on-chip polarizer. The CMOS image sensor was fabricated in an AMS 350-nm OPTO process. The microchip photograph of the CMOS image sensor with an in-pixel polarizer is shown in Figure 3. The on-chip polarizer was created using the metal 1 interconnect on the pixel [29,30]. The pitch of the wire grid polarizer was 700 nm. The pitch of the wire grid was limited by the technology rules of the fabrication house. The spatial resolution of the pixel array was 64 × 128. The pixels were arranged in a 2 × 2 matrix as P0, P1, P2, and P3. Pixel P0 measured the intensity of the incident light. Pixel P1 had a metal grid polarizer oriented with a transmission axis of 0${}^{{}^{\circ}}$, P2 had a polarizer with a transmission axis of 45${}^{{}^{\circ}}$, and P3 had a polarizer with a transmission axis of 90${}^{{}^{\circ}}$. The 2 × 2 pixel array structures with in-pixel polarizers were repeated across the entire imaging array. The pixels with a polarizer of the defined transmission axis allowed the selective transmission of the reflected light whose phase was the same as that of the transmission axis. Therefore, as the phase of the incoming light changed, the received intensity on the pixel with a wire grid followed Malus law. The transmission axis of the external polarizer in front of the light could be manually changed at a step resolution of 1${}^{{}^{\circ}}$. Pixel P0 measured the reflected unpolarized light, while P1, P2, and P3 measured the intensities $I_{0^{{}^{\circ}}}$, $I_{90^{{}^{\circ}}}$, and $I_{45^{{}^{\circ}}}$, respectively. The measured intensity was an average of 100 images captured to remove any temporal noise in the measurement set-up.

The photodiode used in the 3T pixel was n-well/p-sub. The light integrated into the photodiode generated a proportional number of charges which were then converted to voltage in the pixel before being transferred to the column. The analog voltage in the column was passed through a correlated double sampling (CDS) circuit. CDS was used to reduce the thermal noise of the pixel. After the CDS, the analog information was buffered to an external 14-bit ADC. The digital data from the 14-bit ADC were processed using FPGA and saved to a PC for further processing.

**Figure 4:**
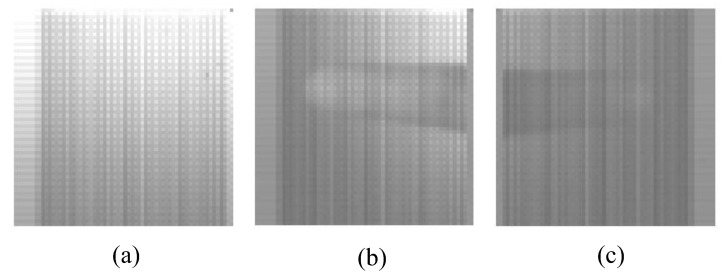
Sample captured images from the experimental set-up. (**a**) without any test object, (**b**) figertip when the incident light is unpolarized, and (**c**) fingertip when the incident light is 45${}^{{}^{\circ}}$ polarized.

Sample monochromatic images captured from the custom CMOS image sensor are shown in Figure 4. The images are raw images from the sensor without any post-processing. The first image (Figure 4a) is a sample image captured from the camera without any test object. The differential intensity due to the presence of a 0${}^{{}^{\circ}}$, 45${}^{{}^{\circ}}$, or 90${}^{{}^{\circ}}$ wire grid polarizer on the pixels is visible. Figure 4b shows a captured image of a fingertip when the light source incident on the fingertip was unpolarized. Figure 4c shows the image captured when the incident light was 45${}^{{}^{\circ}}$ polarized using an external polarizer. The variations in the measured pixel intensities with varying transmission axes allowed phase measurement of the incoming reflected light from the fingertip.

### 3.2. Data Collection

A pilot study was carried out with 12 healthy volunteers (6 males and 6 females). Written consent was obtained from all the volunteers. The volunteers were members of the image sensor lab at IIT Delhi in the age group of 22–30 years. None of the volunteers declared any known cardiovascular diseases. Volunteers who were smokers, pregnant, had hypertension, had any breath-related problems, or had and any form of cosmetics on their fingers were excluded from the study.

All SpO${}_{2}$ measurements were performed in the same place, and the room’s windows were kept closed to minimize the presence of stray ambient light. The experiments were performed at room temperature, or around 30${}^{{}^{\circ}}$. For the experiments, the volunteers were asked to keep their fingers stable without any movement for the entire duration of image capturing. The volunteers were asked to sit comfortably and quietly on a chair in front of the camera system. They were given around 15 min to get accommodated to the test environment before the actual experiments began. The SpO${}_{2}$ measurements were recorded in the sitting position initially in the resting state. The heart rate and SpO${}_{2}$ were also recorded using a commercial pulse oximeter for reference purposes. The measurement data were captured at a sampling rate of 1 s for 15 min. To vary the oxygen saturation in the subjects, acute mild hypoxia is usually used. Hypoxia is an oxygen-derived state of the body resulting in low SpO${}_{2}$ levels [31,32]. A hypoxia state is created using hypoxic air generators. Due to the non-availability of this equipment in the lab, hypoxia-based experiments could not be conducted. To increase their heart rates, the subjects were asked to excite themselves by performing physical activity for 10 min. The images of the fingertips were captured again for SpO${}_{2}$ estimation in the sitting position, and data were sampled for the same duration as the resting state. Since the measurements for the experiments were performed in a completely natural breathing environment while having no physical contact with the subject, ethical approval was not sought.

### 3.3. Data Analysis

The digital data from the 14-bit ADC was processed using FPGA and transferred to a computer through the Cameralink interface. The data from the Cameralink were captured using an in-house custom-developed graphical user interface (GUI) for further processing. The GUI was developed using MATLAB 2021b. The polarized data were obtained from the images captured. The obtained data needed to be filtered for ambient noise reduction and sharpening of the features in the captured signal. In the literature, low-order filters such as the Butterworth, Chebyshev I, and Chebyshev II filters were most commonly used for PPG data processing [33]. Chebyshev I negatively impacts the morphology of the filtered PPG waveform and is therefore often avoided. Chebyshev II filters out the noise interference while maintaining the valuable information in the signal due to its excellent frequency selectivity and no equal ripple in the passband. Filtering has a significant effect on the PPG waveform characteristics [34]. A nonlinear phase response of the applied filters can cause a significant time shift of important feature points in PPG [35]. A zero-phase low-pass filter helps in reducing the time shift in the PPG signal and can achieve more accurate waveform representation but cannot be implemented for real-time filtering [35]. Excessive filtering leads to distortion of the PPG signals and affects its interpretation, whereas insufficient filtering makes the PPG noisy.

The lower-order filters perform better in analyzing biomedical signals [36]. Moreover, the lower-order filters require less computation time, which in turn saves power for wearable devices which are battery-driven. Therefore, in our experiments, for each measurement, 100 images were averaged to perform low-pass filtering of the captured data. Among the captured images, a region of interest (ROI) of 4 × 4 was chosen around the centroid of the finger tip region and averaged. The low-pass filtering helped to smooth out the noise in the captured data while keeping the computational time low.

## 4. Experiment Results

**Figure 5:**
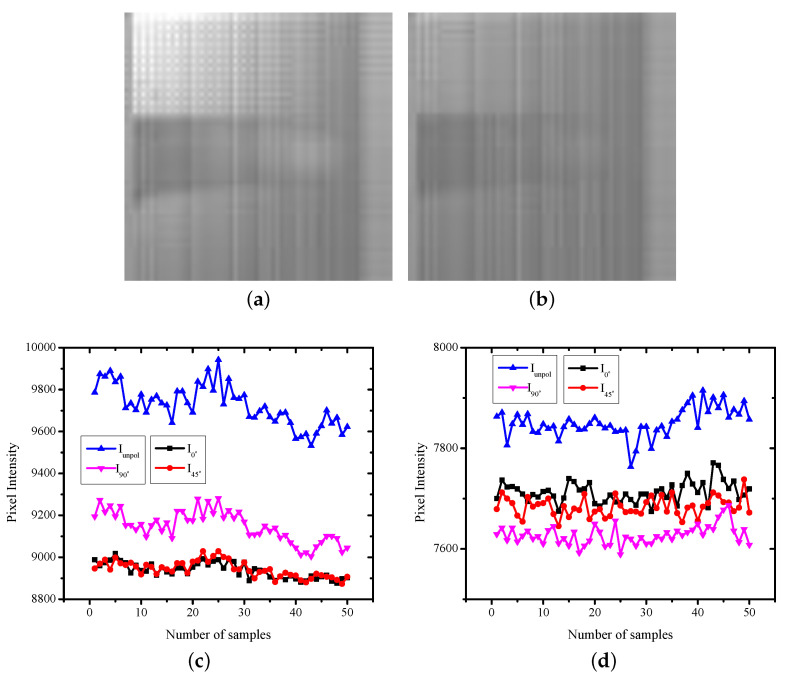
Sample images captured and the intensity variation of pixels with varying transmission axes over time in a resting state, with the incident light being (**a**,**c**) unpolarized or (**b**,**d**) linearly polarized at 45${}^{{}^{\circ}}$.

The polarized images were captured using the measurement set-up shown in Figure 2a. Two sets of images were captured: one with the incident light being unpolarized and the other with the incident light polarized to $45^{{}^{\circ}}$ using an external polarizer, shown in Figure 5a,b, respectively. Figure 5 shows the images captured when the subject was resting. Figure 5c,d shows the variation in the phase of the reflected light over time for unpolarized incident light and $45^{{}^{\circ}}$ linearly polarized light, respectively. As discussed in Section 3.2, each sample was captured in 1 s.

**Figure 6:**
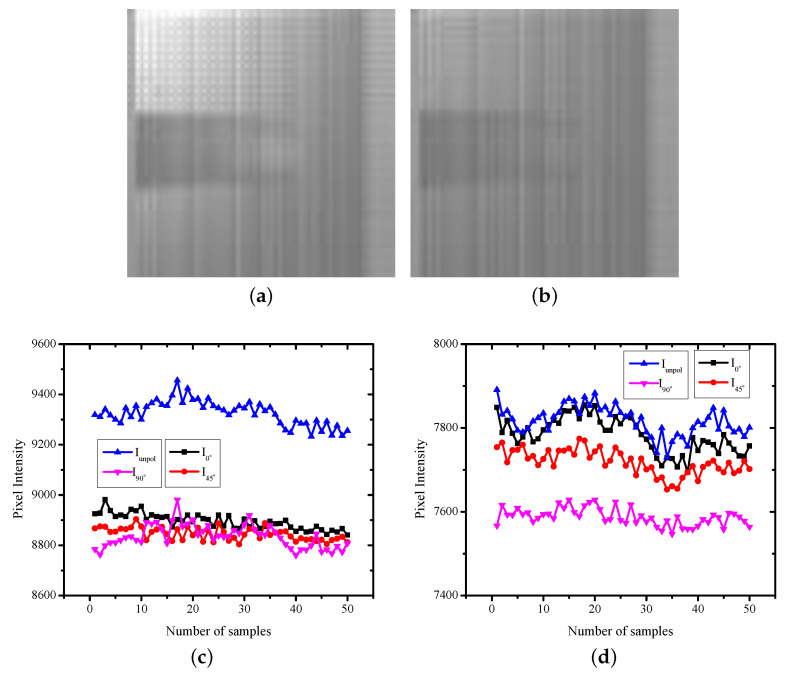
Sample images captured and the intensity variation of the pixels with varying transmission axes over time in an excited state, with incident light being (**a**,**c**) unpolarized or (**b**,**d**) linearly polarized at 45${}^{{}^{\circ}}$.

The imaging sensor consisted of pixels P0, P1, P2, and P3 repeating over the imaging array. The pixel measuring the intensity of the unpolarized light ($I_{unpol}$) was the highest, as there was no attenuation. When the incident light was unpolarized, the intensity of the specularly reflected light from the surface was lower compared with the reflections from the deep layers due to increased depolarization. The received intensity by the unpolarized pixel when the incident light was $45^{{}^{\circ}}$ polarized was smaller compared with when the incident light was unpolarized. This was expected, as the transmitted light when polarized was lower in intensity compared with the unpolarized light. The sample images captured of the same subject in the excited state are shown in Figure 6. In the excited state as well, $I_{unpol}$ was higher when the incident light was unpolarized compared with the incident light being $45^{{}^{\circ}}$ polarized.

When the incident light was unpolarized, the intensity of the specularly reflected light from the surface was lower compared with the reflections from the deep layers due to increased depolarization. For the resting state, $I_{45^{{}^{\circ}}}$ was lower compared with $I_{90^{{}^{\circ}}}$. In the excited state, the increase in HbO${}_{2}$ increased the diffuse reflections, and therefore, $I_{45^{{}^{\circ}}}$ and $I_{90^{{}^{\circ}}}$ tended to be nearly the same. When the transmission axis of the linear polarizer was the same as the phase of the incoming light, the received intensity primarily contained superficial information and specular reflection components. When the transmission axis of the linear polarizer was different from the incoming light phase, the received intensity was primarily from the deep layer of the tissue. For incident light at $45^{{}^{\circ}}$ polarization, the pixel receiving $I_{45^{{}^{\circ}}}$ captured direct reflection coming from the superficial layer and the surface of the skin. Thus, the image corresponding to $I_{45^{{}^{\circ}}}$ was brighter compared with the pixel receiving $I_{90^{{}^{\circ}}}$ phase information.

The pattern of received intensity was the same for the resting state as well as the exciting state. However, for the resting state, the difference between the measured intensity for the $I_{45^{{}^{\circ}}}$ and $I_{90^{{}^{\circ}}}$ pixels was small compared with that for the excited state. The deep layer reflections were lower compared with the specular reflection. This was expected, as the deep layer reflections were highly diffusive since the reflections occurred from arteries that contained pulsatile blood. The diffuse reflections also depended on the blood flow [24], and thus the observed fluctuations represent the pulsation and oxygen saturation.

**Figure 7:**
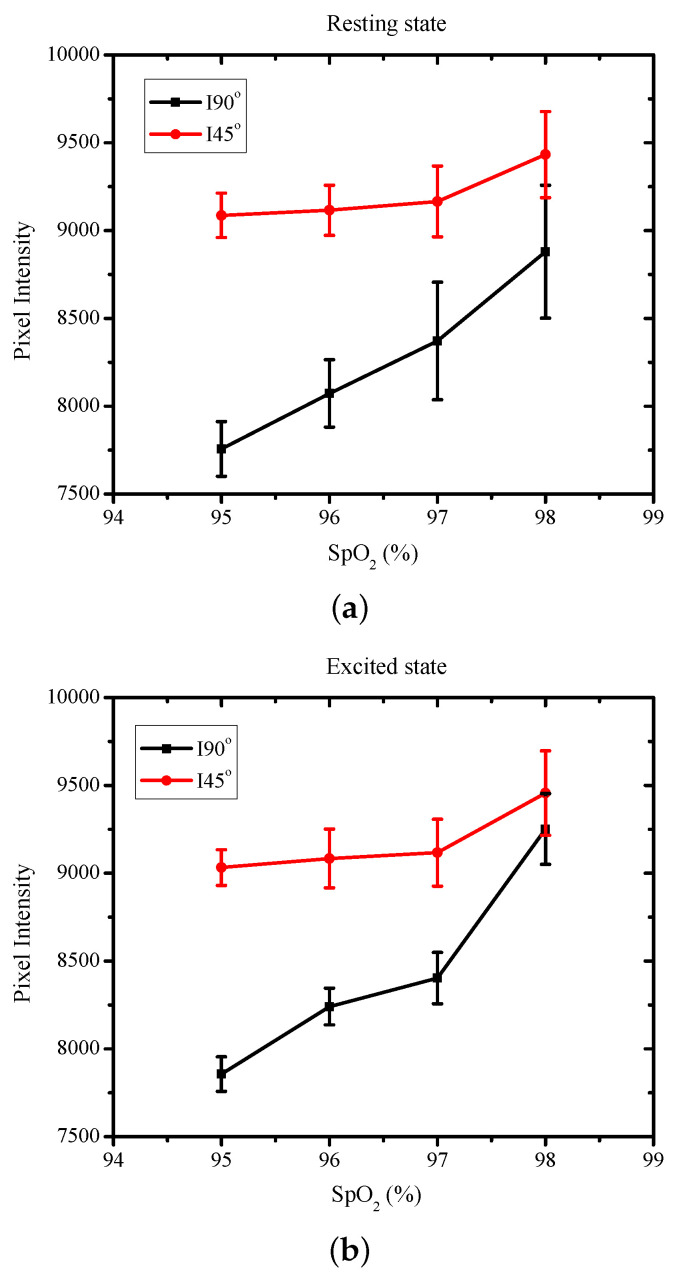
The variations in the pixel intensity $I_{90^{{}^{\circ}}}$ and $I_{45^{{}^{\circ}}}$ for varying SpO${}_{2}$ levels in (**a**) resting state and (**b**) excited state.

The variation in $I_{90^{{}^{\circ}}}$ and $I_{45^{{}^{\circ}}}$ with the varying SpO${}_{2}$ levels for the resting and excited states are shown in Figure 7. The incident light was $45^{{}^{\circ}}$ polarized, and the SpO${}_{2}$ levels of the subjects varied from 95% to 98% during normal breathing patterns. Figure 7a shows the measurements in the resting state. The incident light was $45^{{}^{\circ}}$ polarized, and therefore, the pixel intensity $I_{45^{{}^{\circ}}}$ corresponded to the specular reflection component, while $I_{90^{{}^{\circ}}}$ was from the diffusion from the deep layers. The specular component $I_{45^{{}^{\circ}}}$ was thus higher in intensity compared with $I_{90^{{}^{\circ}}}$. Furthermore, as the specular or superficial reflections were not influenced by the blood flow, the variations in $I_{45^{{}^{\circ}}}$ were also low for increasing SpO${}_{2}$ levels. The variations in $I_{45^{{}^{\circ}}}$ as the SpO${}_{2}$ levels varied from 95% to 98% was only 3.6%. $I_{90^{{}^{\circ}}}$ corresponded to the information from the deep layers and was shown to monotonically vary with variations in the SpO${}_{2}$ levels. The variations in $I_{90^{{}^{\circ}}}$ over the measured range were around 12.5%. Similar behavior was seen in the received intensity in the excited state too, as shown in Figure 7b. As discussed earlier, $I_{45^{{}^{\circ}}}$, as a specular component, was higher than $I_{90^{{}^{\circ}}}$ and showed smaller variations of 4.5%. For the excited state, the variations in $I_{90^{{}^{\circ}}}$ were monotonic as well as larger, being around 15%. The variation in the pixel intensities was higher compared with the resting state.

Figure 7a shows the measurements in the resting state. The incident light was $45^{{}^{\circ}}$ polarized, and therefore, the pixel intensity $I_{45^{{}^{\circ}}}$ corresponded to the specular reflection component, while $I_{90^{{}^{\circ}}}$ was from the diffusion from the deep layers. The specular component $I_{45^{{}^{\circ}}}$ was thus higher in intensity compared with $I_{90^{{}^{\circ}}}$. Furthermore, as the specular or superficial reflections largely were not influenced by the blood flow, the variations in $I_{45^{{}^{\circ}}}$ were also low for increasing SpO${}_{2}$ levels. The variation in $I_{45^{{}^{\circ}}}$ as the SpO${}_{2}$ level varied from 95% to 98% was only 3.6%. $I_{90^{{}^{\circ}}}$ corresponds to the information from the deep layers and was shown to monotonically vary with the variations in SpO${}_{2}$. The variations in $I_{90^{{}^{\circ}}}$ over the measured range were around 12.5%. Similar behavior was seen in the received intensity in the excited state as well, as shown in Figure 7b. As discussed earlier, $I_{45^{{}^{\circ}}}$, being a specular component, was higher than $I_{90^{{}^{\circ}}}$ and showed smaller variations of 4.5%. For the excited state, the variations in $I_{90^{{}^{\circ}}}$ were monotonic as well as larger, being around 15%. The variation in the pixel intensities was higher compared with that in the resting state.

**Figure 8:**
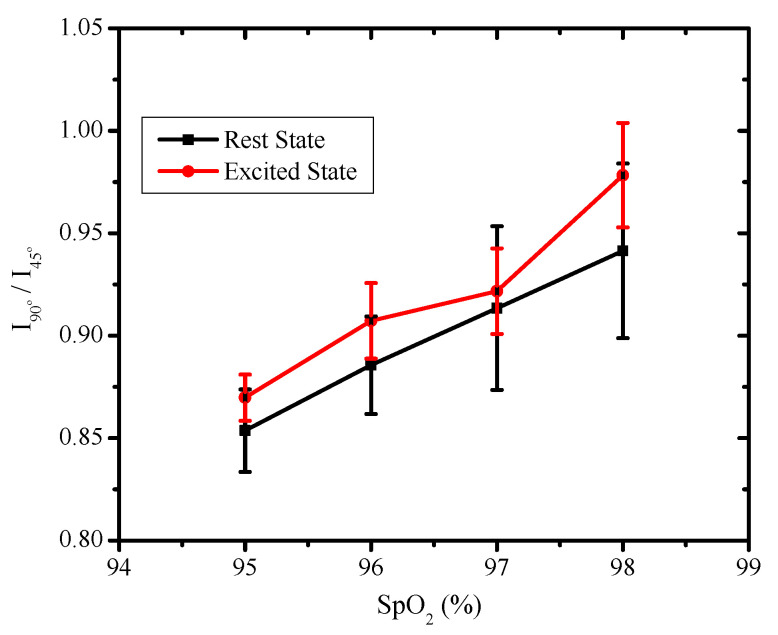
The variation in the $I_{90^{{}^{\circ}}}$/$I_{45^{{}^{\circ}}}$ ratio for varying SpO${}_{2}$ levels.

Figure 8 shows the variation of the ratio of $I_{90^{{}^{\circ}}}$/$I_{45^{{}^{\circ}}}$ for varying SpO${}_{2}$ levels. The ratio $I_{90^{{}^{\circ}}}$/$I_{45^{{}^{\circ}}}$ measures the degree of polarization. The ratio was computed after the individual pixel information was normalized with the intensity information received by the pixel without any wire grid polarizer. The normalization allowed discarding the variations in the measured intensities for individual characteristics such as the color of the skin or other measurement variations, such as background light. A monotonically increasing behavior was observed in the ratio $I_{90^{{}^{\circ}}}$/$I_{45^{{}^{\circ}}}$ for both the resting and excited states. The excited state showed higher variations compared with the resting state. The higher variations were due to increased levels of HbO${}_{2}$ and heart rate. Thus, a higher $I_{90^{{}^{\circ}}}$/$I_{45^{{}^{\circ}}}$ ratio corresponded to increased SpO${}_{2}$ levels and increased heart rate. The measured heart rate and SpO${}_{2}$ using the commercial oximeter are also shown in Table 1. The increase in the variations in the received pixel intensities for the excited state directly corresponded to the increase in the heart rate to maintain the SpO${}_{2}$ levels.

A comparison of the proposed method with similar SpO${}_{2}$ measuring devices available in the literature is shown in Table 2. In [24], $I_{deep}$ and $I_{superfical}$ were obtained using external polarization filters. The light from a DC light source was linearly polarized using an external filter. The polarized light was incident on the fingertip, and the reflected light was transmitted through another external filter before being detected by the camera system. The use of external filters introduced air gaps between the polarizer or analyzer and the camera system. These air gaps depolarized the reflected light, resulting in a larger variance in the measurement error and lower accuracy. To reduce the measurement errors, a custom CMOS image sensor with an in-pixel wire grid polarizer was used in this work. The use of an in-pixel wire grid polarizer prevented depolarization of the backscattered light. The sensitivity of the variation in the received intensities can be further increased by increasing the extinction ratio of the wire grid polarizer created on the pixel. The extinction ratio can be increased by reducing the pitch of the wire grid polarizer by using a lower technology node to fabricate the custom CMOS image sensor.

The monotonic variations in the $I_{90^{{}^{\circ}}}$/$I_{45^{{}^{\circ}}}$ ratio with the SpO${}_{2}$ level and heart rate provide a simpler instrumentation solution for non-contact and noninvasive measurements of the heart rate and SpO${}_{2}$ level. The measurements are obtained with white light, and thus ambient light can be used. This eliminates the need for an additional light source in the measurement set-up. The use of the ratio to determine the resting or excited state of the SpO${}_{2}$ level of an individual is independent of the position of the fingertip and the motion of the subject to a large extent. The noise in the measurements can be suppressed by efficient filtering algorithms. The filtering effects on the phase noise measured in the polarized signals over time will be explored in future works. The low computational requirements make the proposed design achieve real-time measurements of the SpO${}_{2}$ levels and enhance basic health care.

## 5. Conclusions

A rapid non-contact and noninvasive measurement of SpO${}_{2}$ is highly useful in quick analysis of the health status of an individual. The proposed method presents a polarized, imaging-based, cost-effective, accurate, simple, and real-time solution for estimating the resting or excited state of the heart rate as well as the SpO${}_{2}$ level. The proposed method uses ambient white light and a ratio of measured pixel intensities with varying transmission axes. The embedded polarizer is implemented using a wire grid and integrated on top of the pixel. The proposed solution is thus a compact system, eliminating the use of external polarizers and dedicated light sources.

The wire grid polarizers on the pixel help determine the intensity corresponding to a certain phase and thus help in separating the reflected light component from the specular and deep tissue reflections. The ratio of these components is shown to monotonically increase with varying SpO${}_{2}$ levels and heart rate. The experimental results obtained were benchmarked with commercially available oximeters, and thorough statistical analysis was performed. The experimental results were consistent with the reference values. The excited state ratio was higher compared with the resting state due to increased HbO${}_{2}$ level, which depolarized the light further. This method thus quickly determines the nature of the heart rate for immediate medical attention. The polarization-based imaging techniques possess great potential in making cost-effective and compact integrated devices for biomedical applications.

## Figures and Tables

**Table 1:** Measured heart rate and SpO${}_{2}$ levels for the resting and excited states versus the $I_{90^{{}^{\circ}}}$/$I_{45^{{}^{\circ}}}$ ratio obtained from the measurements. The heart rate and SpO${}_{2}$ in columns I, II, and III are reference measurements from the commercial oximeter.

H.R.	H.R.	SpO${}_{2}$	$I_{90^{{}^{\circ}}}/I_{45^{{}^{\circ}}}$	$I_{90^{{}^{\circ}}}/I_{45^{{}^{\circ}}}$
(Resting)	(Excited)		(Resting)	(Excited)
(I)	(II)	(III)	(IV)	(V)
65	85	95	0.853	0.869
68	93	96	0.885	0.907
74	109	97	0.913	0.921
82	114	98	0.941	0.978

**Table 2:** Comparison with the state of the art.

	[22]	[15]	[24]	Proposed Work
Algorithm used	Radially polarized light from LED and an external camera system used for SpO${}_{2}$ measurements	Smartphone camera is used for capturing reflectance image while flashlight is used as illumination	Ratio of phase information from deep and superficial backscattering is used to estimate SpO${}_{2}$	In-pixel wire grid polarizers are used to compute the ratio of phase information from deep and superficial backscattering
Light sources or wavelength used	One	One	One	One
Optical test bench	Complex due to use of collimated optics along with external polarizers	Simple, with no optical test bench used. Does not use phase information.	Complex due to use of polarizer and analyzer with manual varying of the transmission axis of the filters	Simple, with no manual variations of the transmission axis of the filter required
Sampling time	16 s	2 s	3.4 s	1 s
Computational complexity	Simple. The mean intensity at different polarization states is used to compute the ratio of polarization states.	Complex. Ratio of the green and red channel of the mobile phone camera is used along with appropriate weights in the calibration.	Simple. The ratio of intensities for two phases is used with normalization.	Simple. The ratio of intensities for two phases is used with normalization.
Ambient noise reduction	The polarizer filters in the optical test bench reduce specular reflection	The polarizer (near flashlight) and analyzer (near camera) were orthogonally oriented to minimize the specular reflection	The external polarizer filter near the camera reduced the specular reflection	The in-pixel embedded polarizer reduces the specular reflection
Accuracy	Not provided	2–3%	The ratio of intensities of two phases is shown to be linear	The ratio of intensities of two phases is shown to be linear
Method used to vary oxygen saturation	Normal breathing conditions while sitting down were used for measurements	Medical grade manual pressure cuff was used to vary the oxygen saturation	The subject was asked to hold his or her breath for some time	The subject was asked to perform physical activity for 10 min
Number of test subjects	6	5	15	12
